# Apparently isolated CNS involvement in Erdheim-Chester disease: Case report

**DOI:** 10.1016/j.radcr.2023.09.033

**Published:** 2023-10-07

**Authors:** Giuseppe Romano, Mario Cirillo, Simona Bonavita, Giuseppe Toro, Andrea Di Pietro, Luigi Lavorgna, Elisabetta Maida, Francesca Pagliuca, Fabrizio Urraro, Cinzia Coppola, Giacomo Lus, Elisabetta Signoriello

**Affiliations:** a Second Division of Neurology, University of Campania “Luigi Vanvitelli”, Naples, Italy; b MRI Research Center SUN-FISM, University of Campania “Luigi Vanvitelli”, Naples, Italy; c Department of Medical and Surgical Specialties and Dentistry, University of Campania “Luigi Vanvitelli”, Naples, Italy; d First Division of Neurology, University of Campania “Luigi Vanvitelli”, Naples, Italy; e Pathology Unit, Department of Mental and Physical Health and Preventive Medicine, “Luigi Vanvitelli”, Naples, Italy; f Department of Precision Medicine, University of Campania “Luigi Vanvitelli”, Naples, Italy

**Keywords:** Erdheim-Chester, MRI, Bone lesions

## Abstract

We present the case of a 48-year-old-woman with apparently isolated central nervous system Erdheim-Chester disease characterized by brainstem involvement. Erdheim-Chester disease is extremely rare and multisystem impairment should always be sought in the suspicion of such pathology.

## Introduction

We present an unusual case of a 48-year-old woman with apparently isolated central nervous system (CNS) Erdheim-Chester (ECD) disease, displaying neurological symptoms in the absence of any systemic manifestations. ECD is an extremely rare condition, and consideration of potential multisystem involvement is crucial when suspecting this pathology. The patient, after proper informed consent, has allowed the disclosure of her clinical case

## Case report

**Fig. 1:**
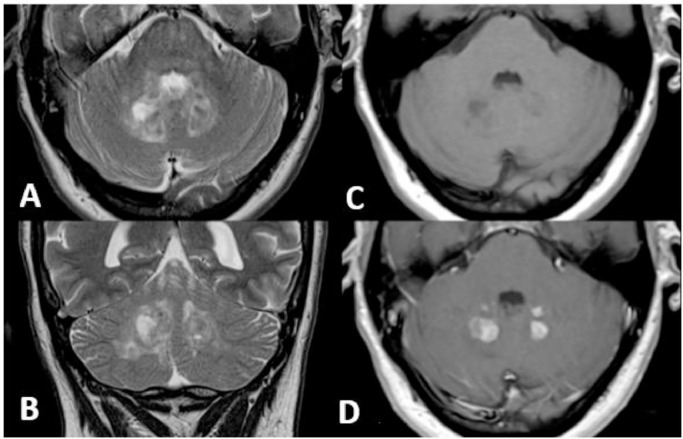
(A-B) T2 sequence showed hyperintensity of dentate nuclei with calcifications (top in axial; bottom in coronal). (C-D) T1 sequence in axial without mdc showed hypointensity of dentate nuclei more evident on the right than on the left; postcontrast T1 sequence in axial showed intense mdc nodular uptake of the dentate nuclei.

**Fig. 2:**
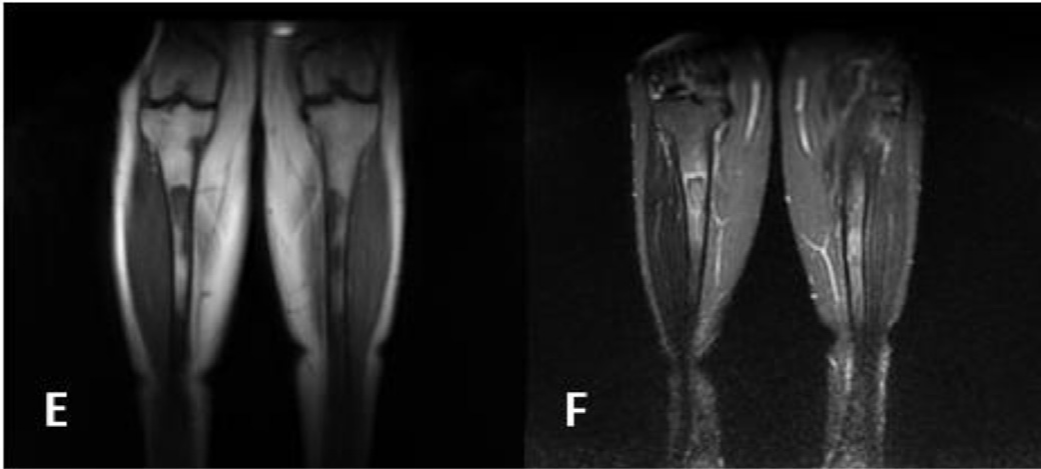
(E-F) T1 sequence showed hypointense osteosclerotic lesions of tibial diaphysis, bilaterally; T2 sequence showed the hyperintense rim of the same lesions.

**Fig. 3:**
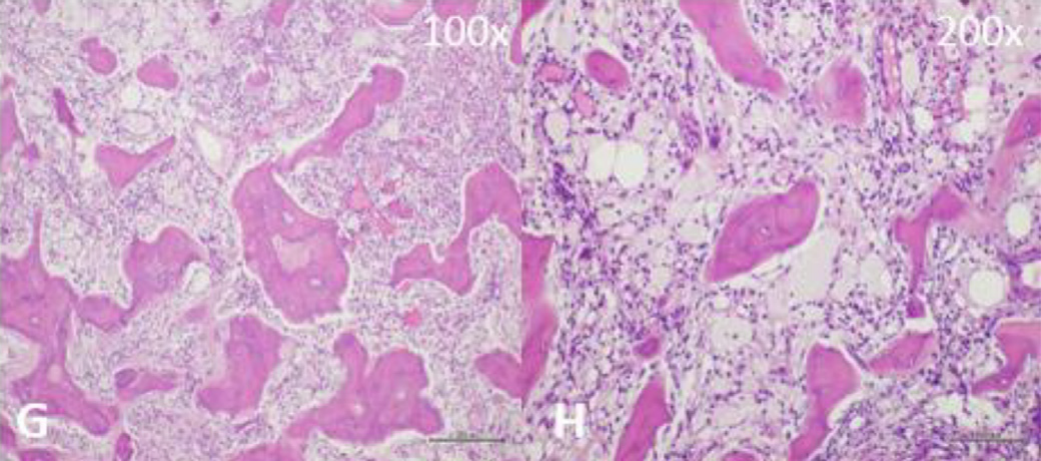
(G-H) Bone biopsy showing infiltration of foamy histiocytes in a mildly fibrotic background (Hematoxylin and Eosin stain; original magnification: 100× and 200×).

A 48-year-old woman presented with 2 years of progressive clumsiness in her right hand, accompanied by dysarthria, dysphagia (especially for liquids), and an unsteady gait. Her past medical history was unremarkable. A general objective examination revealed multiple abdominal and dorsal lipomas. The neurological assessment indicated a slightly ataxic gait with tandem difficulty, left inferior facial paresis, hyper-reflexia in both upper and lower limbs, dysarthria causing slurred speech, and mild clumsiness during cerebellar tests (diadochokinesia, finger-to-nose, and knee-heel test), along with abnormal primitive reflexes. Brain MRI without contrast enhancement displayed symmetric hyperintensity of the cerebellar peduncles and dentate nuclei bilaterally, accompanied by calcifications (Figs. 1A and B). Considering the clinical history, lipomas, and MRI findings, Xanthogranulomatosis Cerebrotendinous, and systemic diseases were ruled out. Hematological, biochemical, autoimmune, neo, and paraneoplastic screenings yielded normal results. Subsequent tests for hypothalamic-pituitary hormones and plasma cholestanol levels fell within the normal range. Genetic testing for sterol 27-hydroxylase gene (CYP27A1) mutations returned negative results. A subsequent brain MRI with contrast enhancement exhibited enlarged hyperintense T2/FLAIR lesions in the cerebellar peduncles and dentate nuclei bilaterally, along with intense homogeneous gadolinium enhancement in T1-weighted images (Figs. 1C and D). Additionally, 2 symmetrical slightly hyperintense periorbital neoformations were noted. Following this new information, and suspecting histiocytosis, cardiology evaluation and a transthoracic echocardiogram ruled out cardiac involvement. A thoraco-abdominal CT scan with contrast excluded aortic and periaortic involvement, perirenal fat infiltration, and retroperitoneal fibrosis. Limb radiographs displayed bilateral, symmetrical cortical thickening of the humeral and femoral diaphysis with periosteal reaction. Consequently, a whole-body MRI revealed osteosclerotic lesions in the left humerus, distal epiphyses of both femurs, and the bodies of both tibias. These lesions appeared hypointense in T1 and T2 weighted images, with a hyperintense rim (Figs. 2E and F). The patient underwent a CT-guided bone biopsy, which revealed fibrosis and a foamy histiocytic infiltrate with scarce lymphocytes; no Touton giant cells were observed. Immunohistochemical analysis of foamy histiocytes showed positive immunostaining for CD68 and fascin, while CD1a and S100 were negative. BRAF V600E mutation-specific immunostaining was also negative (Figs. 3G and H). The diagnosis of Erdheim-Chester disease was established through a combination of clinical, instrumental, and histological data.

## Discussion

ECD is a rare, multisystem disorder with approximately 1500 cases reported in the literature. The typical age of onset falls between 40 and 56 years, with a male-to-female ratio of 2.3:1 [[1], [2], [3]]. ECD is classified as a neoplastic disease, and mutations in the mitogen-activated protein kinase pathway have been identified, leading to the expansion and accumulation of monocyte-derived macrophages [1]. The BRAF V600E point mutation is found in about half of the cases, and treatment with Vemurafenib has been approved for these cases [4]. The definitive diagnosis of ECD requires lesion biopsy. Histological examination reveals foam-activated histiocytes within a fibrotic context, occasionally accompanied by Touton's giant cells. Immunohistochemical examination shows positive CD68 and CD163 staining in histiocytes, while CD1a staining is negative, distinguishing them from Langerhans cells observed in Langerhans cell histiocytosis [[2], [3]]. Clinical manifestations and disease progression vary based on the systems involved. The skeletal system (90%) is the most commonly affected, with lower limb involvement featuring osteosclerotic lesions and associated pain in half of the cases. However, CNS involvement is associated with a worse prognosis and high mortality [[2], [5]]. CNS involvement occurs in around half of the cases during the disease course, with pyramidal and cerebellar manifestations being the most frequent. Other possible symptoms include seizures, headaches, neuropsychiatric symptoms, cognitive deficits, sensory disturbances, and cranial nerve palsies. Conversely, CNS involvement in isolation, without systemic signs or symptoms, is rare [5]. Three MRI patterns are described: vascular, tumor, and neurodegenerative. An overlapping pattern can also be detected, particularly with typical features of degenerative and tumor forms. Atypical presentations like limbic encephalitis, pseudo-CLIPPERS, and pseudo-multiple sclerosis are rare [6]. In our case, the patient presented solely with neurological signs/symptoms, making the diagnosis challenging. However, given the highly suggestive clinical and MRI features, along with the previously described overlapping tumoral and neurodegenerative pattern, the diagnostic process had dual aims. Firstly, it aimed to exclude various pathologies that could mimic the clinical-radiological presentation. Consequently, differential diagnoses included autoimmune, neoplastic-paraneoplastic, and genetic diseases such as Cerebrotendinous Xanthogranulomatosis and Langerhans cell histiocytosis; common demyelinating diseases were ruled out due to unusual MRI features. Secondly, considering the absence of systemic symptoms, identifying secondary disease localizations through various imaging tests, suitable for biopsy confirmation, was essential. While X-rays were inconclusive for potential secondary bone localizations, whole-body MRI played a critical role in ensuring an accurate diagnostic framework.

## Conclusions

ECD, an uncommon histiocytic neoplasm, presents diverse clinical manifestations, posing significant diagnostic challenges, particularly due to its rarity. In our case, the absence of systemic symptoms led to a diagnostic focus on neurological manifestations, supported by MRI findings.

## Patient consent

Written informed consent for the publication of this case report was obtained from the patient.
